# Clinical Application of Proximal Arch Cannulation in the Surgical Treatment of Acute Type I Aortic Dissection

**DOI:** 10.7759/cureus.37214

**Published:** 2023-04-06

**Authors:** Asfandyar Khan, Chaoen Luo, Fan Hu, Peiyun Zhang, Chaozhong Long, Yaoguang Feng, Zhengwen Lei, Tajallah Khan

**Affiliations:** 1 Department of Cardiothoracic Surgery, The First Affiliated Hospital, Hengyang Medical School, University of South China, Hengyang, CHN

**Keywords:** true and false lumen, perfusion strategy, morphological analysis, supraaortic arch cannulation, acute debakey type i aortic dissection

## Abstract

Objective

The goal is to determine the best location for inserting a catheter into the aortic arch of patients with a certain type of aortic dissection (DeBakey type I) by analyzing images of the patient's aortic arch before surgery. This analysis will take into account the shape and structure of the patient's aortic arch to find the most optimal location for cannulation.

Methods

A retrospective analysis was conducted on 100 patients with acute DeBakey type I aortic dissection diagnosed between January 2021 and February 2023, utilizing the Carestream medical imaging software Image Suite V4 (New York, USA). The study included 67 cases that underwent surgery and 33 cases that did not. The study aimed to evaluate the optimal intubation position on the patient's aortic arch by analyzing the true and false lumen classification, true and false lumen area, and hematoma thickness on the patient's aortic arch, as observed in the aortic computed tomography angiography (CTA) conducted upon admission.

Results

The vascular axis analysis showed a significant difference in the true lumen area among the three regions that were examined (P < 0.001). Zone 1 had a larger true lumen area of 6.40 ± 2.71 cm^2^ compared to zone 2 with 5.75 ± 2.13 cm^2^ and zone 3 with 4.85 ± 1.70 cm^2^, as determined by statistical analysis.

In addition, the statistical analysis of hematoma thickness in the three regions where cannulation can be performed revealed a significant difference among the three groups (P = 0.027). Further analysis showed that there was no significant difference between zone 1 and zone 2 (P = 1.000), a significant difference between zone 1 and zone 3 (P < 0.046), and no significant difference between zone 2 and zone 3 (P = 0.080). The difference between zone 1 false lumen thickness of 1.55 ± 0.51 cm and zone 3 false lumen thickness of 1.33 ± 0.55 cm was found to be small.

Conclusion

Cannulation of the aortic arch is a common strategy used in cardiac surgery. Accurate cannulation is critical to the success of the procedure. The use of CTA provides valuable guidance for the cannulation procedure. A thorough examination of CTA and precise measurement of relevant parameters can help guide the surgeon to determine the optimal cannulation site. The study found that zone 1 of the aortic arch has the largest area and is the most suitable for cannulation, in accordance with the physiological characteristics and surgical practices of a surgeon. Furthermore, cannulation of the aortic arch has been found to be a safe and effective strategy for cannulation. Overall, careful examination of CTA and accurate measurement of relevant parameters can have a significant guiding effect on the cannulation of the aortic arch, which can lead to improved outcomes in cardiac surgery.

## Introduction

Aortic dissection is a severe cardiovascular illness with a grave prognosis and high mortality. In cases of acute DeBakey type I aortic dissection, the mortality rate increases by 1-2% per hour without treatment within 24 hours of onset, and exceeds 70% within one week of onset. Even chronic type I aortic dissection poses a risk of death due to complications such as aortic rupture and organ failure [1]. In the medical community, there is a consensus among experts both domestically and internationally that once diagnosed, emergency surgery is the recommended immediate treatment for type I aortic dissection. This consensus is supported by the 2014 European Heart Association guidelines for the diagnosis and treatment of aortic diseases, which classify surgical intervention as a class IB recommended indicator for the treatment of this condition [2]. In surgical interventions for type I aortic dissection, the choice of cannulation and perfusion strategies during extracorporeal circulation is essential for ensuring surgical safety and optimizing patient outcomes [3]. Currently, there exist several perfusion strategies for surgical interventions in aortic dissection, including axillary artery cannulation for perfusion, femoral artery cannulation for perfusion, and combined femoral-axillary dual perfusion, among other techniques [4,5], which have significantly advanced the development of perfusion technology in surgical interventions for aortic dissection. Despite their benefits, various perfusion catheterization strategies have inherent limitations. For instance, retrograde perfusion via the femoral artery carries the risks of false lumen perfusion, inadequate organ perfusion, and cerebral embolism [6,7]. On the other hand, the right-sided axillary artery cannulation technique poses certain challenges, including extended procedural duration, potential brachial plexus injury, insufficient perfusion, and difficulties in monitoring right radial artery blood pressure during antegrade cerebral perfusion [4]. The optimal perfusion strategy for cannulation remains a topic of debate in the medical community. Consequently, there is a need to investigate novel approaches to enhance current perfusion techniques. Japanese researchers have proposed that cannulation perfusion of the ascending aorta represents a secure and efficacious method, and this facility has subdivided the aortic arch into three distinct regions suitable for cannulation. This research study enrolled 100 patients with acute DeBakey type I aortic dissection who underwent preoperative treatment at the investigated center. The patients were assessed using preoperative aortic computed tomography angiography (CTA) images to evaluate the relationship between the true and false lumens, as well as the cross-sectional area of the aorta, true lumen, and false lumen. The depth and angle of the puncture needle, along with the surgeon's position, were also considered. Through a combination of surgical practice and analysis, this study aimed to identify the most appropriate cannulation perfusion location within the three areas of the aortic arch. Ultimately, this research provides a new theoretical and practical framework to enhance perfusion strategies for aortic dissection surgery.

## Materials and methods

General information

In this study, we conducted a retrospective analysis of 100 patients (79 males and 21 females) diagnosed with acute DeBakey type I aortic dissection at our center between January 2021 and February 2023. We collected data such as age, sex, past medical history, and ejection fraction from their medical records. We used Carestream medical imaging software Image Suite V4 (New York, USA) to analyze the relationship between the true and false lumens of the aortic arch, the cross-sectional area of the aorta, the cross-sectional area of the true lumen, the cross-sectional area of the false lumen, and the depth of the puncture needle based on CTA examination images of the aorta. Of the 100 patients, 67 underwent catheter cannulation of the aortic arch, and we collected relevant data to establish a database.

Inclusion and exclusion criteria

The study was conducted at the First Affiliated Hospital of University of South China between January 2021 and February 2023, involving 100 patients diagnosed with acute DeBakey type I aortic dissection. The study focused on conducting a morphological analysis of the aortic arch in these patients.

The study excluded patients with non-acute DeBakey I aortic dissection, as well as patients who did not undergo preoperative CTA or had a prior history of cardiac or vascular surgery.

This study has received ethical approval from the Ethics Committee of the First Affiliated Hospital of University of South China.

Experimental method

**Figure 1:**
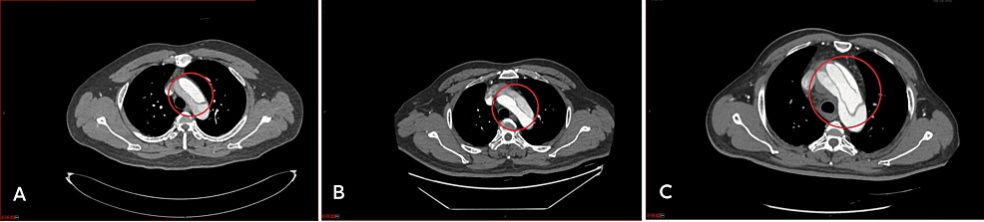
Types of the true and false lumen of the aortic arch (A) True lumen-false lumen (T, F). (B) False lumen-true lumen (F, T). (C) False lumen-true lumen-false lumen (F, T, F).

**Figure 2:**
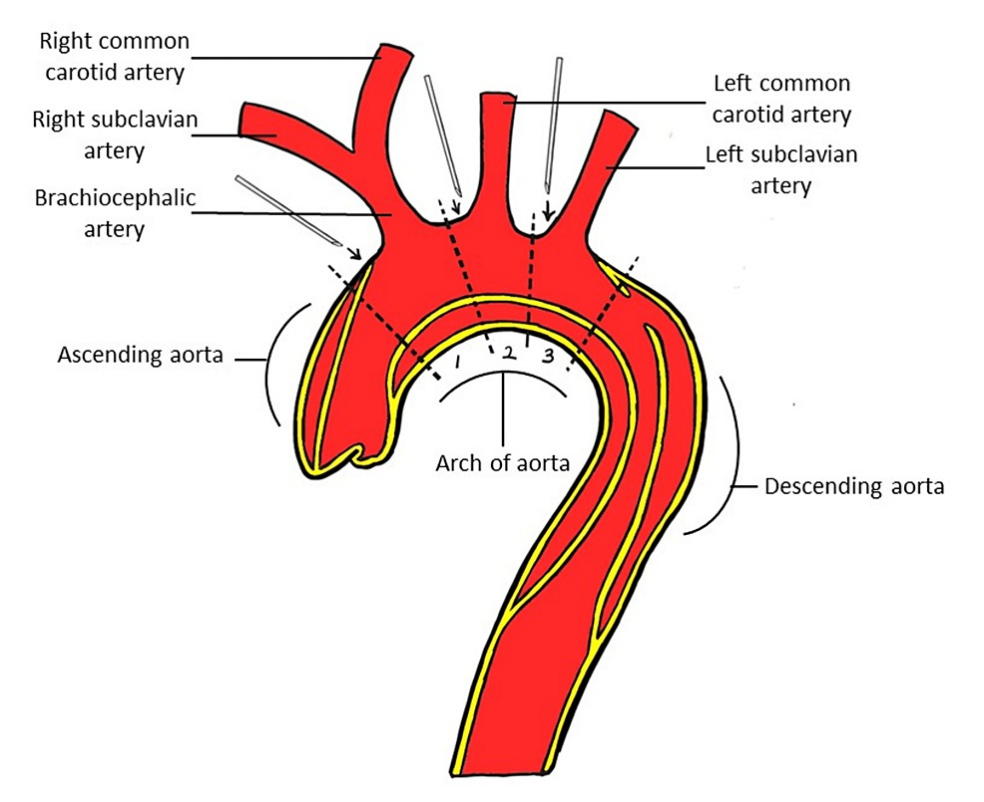
Supra-aortic cannulation zones Zone 1, zone 2, and zone 3. Available as a choice of three cannulation areas on the aortic arch. Figure created by Asfandyar Khan.

**Figure 3:**
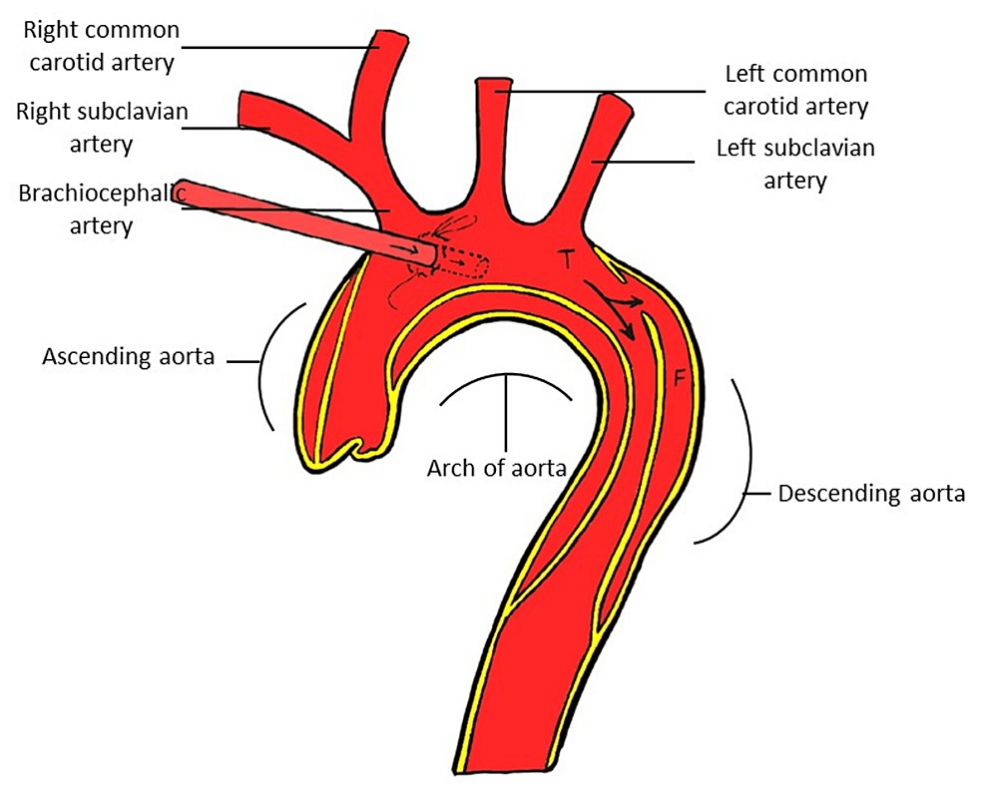
Diagram of cannulation perfusion in zone 1 of the aortic arch Figure created by Asfandyar Khan.

**Figure 4:**
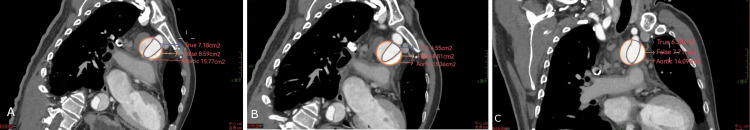
Areas of true and false lumens in three zones on the aortic arch (A) Zone 1, (B) zone 2, and (C) zone of true and false lumens in zone 3.

**Figure 5:**
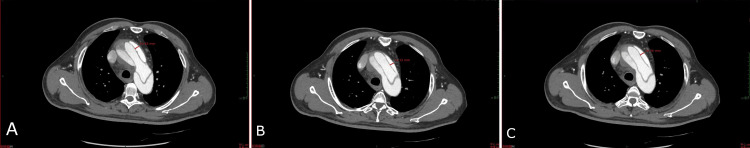
The thickness of true and false lumen hematoma on the aortic arch and the depth of puncture (A) Zone 1, (B) zone 2, and (C) zone 3 hematoma thickness.

The study involves the classification of the spatial orientation of the true and false lumens of a patient's aortic arch into three types based on the chest bone serving as the upper boundary of the spine. The first type has the true lumen positioned above the false lumen (T, F), the second type has the false lumen above the true lumen (F, T), while the third type has the false lumen situated between two true lumens (F, T, F), as shown in Figure 1. The aortic arch region is divided into three zones based on specific anatomical landmarks, with zone 1 corresponding to the distal end of the ascending aorta up to the trunk of the brachial artery and the midpoint of the left common carotid artery, zone 2 corresponding to the trunk of the brachial artery and the midpoint of the left common carotid artery up to the midpoint of the left common carotid artery and the left subclavian artery, and zone 3 corresponding to the segment of the aortic arch from the midpoint of the left common carotid artery and the left subclavian artery up to the beginning segment of the descending aorta. These zones are illustrated in Figure 2. Cannulation perfusion in zone 1 of the aortic arch is illustrated in Figure 3. The areas of the true lumen, false lumen, and total vascular area are measured and recorded in zone 1, zone 2, and zone 3 of the aortic arch, as depicted in Figure 4. The thickness of the thrombus near the chest bone is measured from the vascular axis of the aortic arch, as shown in Figure 5. The human body axis is defined as the patient lying in a supine position with the chest bone serving as the upper spine, and the positional relationship between the true and false lumens of the patient's aortic arch is observed. The vascular axis is defined as the axis formed by the direction of the patient's aortic arch vessel. The standard true lumen, false lumen area, and thrombus thickness are measured in the cross-sectional plane of this axis. However, the thrombus thickness is not measured and statistically analyzed in the true lumen-false lumen type where the true lumen is situated above, as this type is more suitable for cannulation. The zones and their corresponding landmarks are illustrated in Figure 2.

Surgical method

Following the successful administration of anesthesia, the patient was placed in a supine position and an esophageal ultrasound probe was inserted. The chest was disinfected and suspended, and a midline incision was made to open the chest. The brachiocephalic trunk, left common carotid artery, and left subclavian artery were sequentially exposed and tied off. The patient was given systemic heparinization at a dose of 3 mg/kg. A 3-0 prolene purse string suture was made on the aortic arch, which was then punctured with a disposable minimally invasive puncture needle from Shenzhen, China. The correct placement of the needle and guide wire in the aortic arch's true lumen was confirmed using the esophageal ultrasound probe. After removing the needle, a catheter was inserted into the aortic arch through the guide wire. The catheter was connected to extracorporeal circulation after air removal, and the infusion pressure was tested. After achieving normal infusion pressure, the aortic catheter was secured, and a single chamber catheter was inserted into the right atrium to establish extracorporeal circulation and parallel circulation for cooling. A left heart drainage tube was inserted through the right upper pulmonary vein. The proximal ascending aorta was blocked, and ice chips were placed on the heart's surface. Ventricular fibrillation was induced, and the ascending aorta was longitudinally cut. The myocardial protection solution was infused sequentially through the openings of the left and right coronary arteries, and successful aortic infusion was obtained. The diseased part of the ascending aorta (sinotubular junction-proximal ascending aorta) was removed, and the aortic valve and sinus were explored. Proximal anastomosis of the aorta was formed using "sandwich" suturing with 5-0 prolene. When the nasopharyngeal temperature reached 25°C, the branches of the aortic arch were sequentially occluded (brachiocephalic trunk, left common carotid artery, and left subclavian artery), and the branches of the aortic arch were infused with 10 ml/kg of whole brain infusion. The aortic arch arterial infusion tube was clamped, the lower body blood supply was stopped, and the aortic arch was cut and removed in order. A stent was placed in the descending aorta, and the distal end of the four-branch artificial blood vessel was sutured to the artificial blood vessel and the descending aorta of the stent with 3-0 prolene. The three branches of the four-branch artificial blood vessel and the proximal end of the artificial blood vessel were clamped, and the second infusion tube of the extracorporeal circulation was connected to the fourth branch of the artificial blood vessel. The lower body blood supply was restored, and the proximal end of the artificial blood vessel was sutured to the proximal autologous blood vessel with 4-0 prolene. The heart was rewarmed and restored automatically, and the three branches of the artificial blood vessel were sutured to the left common carotid artery, left subclavian artery, and brachiocephalic trunk to restore blood supply to the head. After repaying the oxygen debt fully, the extracorporeal circulation was gradually withdrawn when the temperature reached 36°C, and the chest was closed after stopping the bleeding and ensuring stable blood pressure and heart rate.

Statistical methods

The statistical analysis was conducted using SPSS version 26 software (IBM Corp., Armonk, NY). The measurement data were expressed as mean ± standard deviation (X ± S) and were analyzed for normal distribution. If the data met the criteria of independence, quantification, normal distribution, and homogeneity of variance, group comparison was performed using multiple-sample variance analysis. In cases where variance was not homogeneous, Welch's test was utilized.

## Results

Characteristics of true and false lumen classification in acute DeBakey type I aortic dissection patients

This study comprised a cohort of 100 patients diagnosed with acute DeBakey type I aortic dissection, with a mean age of 52.99 years. Among them, 79 were male, 27 had a history of smoking, 64 had hypertension, 10 had diabetes, 13 had coronary artery disease, and six had a history of central/peripheral vascular disease.

The study assessed the relationship between the true and false lumen based on the morphological characteristics of the patients' aortic arch, from the top to the bottom of the sternum. The results showed that among the analyzed cases, 38% had a true lumen-false lumen type, 30% had a false lumen-true lumen type, and 32% had a false lumen-true lumen-false lumen type, as indicated in Figure 1 and Table 1.

**Table 1:** General information about the patients and the relationship between true and false lumen positions on the arch

General	Patients (n = 100)
Age	52.99 (26-80)
Male/female	79/21
Smokers	27 (100)
Hypertension	64 (100)
Diabetes	10 (100)
Coronary heart disease	13 (100)
Central peripheral vascular disease	6 (100)
Preoperative ejection fraction	59.36% (34%-77%)
True lumen-false lumen (T, F)	38 (100)
False lumen-true lumen (F, T)	30 (100)
False lumen-true lumen-false lumen (F, T, F)	32 (100)

Areas of true and false lumens and puncture needle depths in the three zones on the aortic arch

In this experiment, three accessible zones of the aortic arch were measured based on the morphological characteristics of the patient's aortic CTA arch using human axis and vascular axis measurements. Statistical analysis revealed that there was a significant difference in the true lumen area overall (F' = 13.298, P< 0.001), with no significant difference between zones 1 and 2 (P = 0.12), but significant differences between zones 1 and 3 (P < 0.001) and zones 2 and 3 (P = 0.013). There was also a significant difference in the false lumen area overall (F' = 36.152, P < 0.001), with significant differences between zones 1 and 2 (P < 0.001), zones 1 and 3 (P < 0.001), and zones 2 and 3 (P < 0.001). Lastly, there was a significant difference in the total area overall (F' = 80.575, P < 0.001), with significant differences between zones 1 and 2 (P < 0.001), zones 1 and 3 (P < 0.001), and zones 2 and 3 (P < 0.001). The experiment showed that although there were no significant differences between the true lumen area in zone 1 and zone 2, zone 1 had a relatively larger true lumen area and was located closer to the main incision, without any obstructions from organs such as the mediastinum, pulmonary tissue, or ribs. Additionally, the cannulation length into the aortic arch was around 5-6 cm, and the insertion part was entirely in the aortic arch, which resulted in good perfusion of the three branches in the arch. Therefore, based on these findings, zone 1 was determined to be the most suitable area for cannulation among the three zones.

In this study, patients with aortic dissection and a true-false lumen type (Figure 1A), where the true lumen is located above and the false lumen below, can undergo direct puncture and catheter insertion during surgery. Out of the 100 patients enrolled, 38 of this type were not included in the measurement of false lumen hematoma thickness and comparison of puncture depth. For the remaining 62 patients with a false-true or false-true-false lumen type, three zones for catheter insertion were defined and compared. The results showed statistically significant differences between the three groups (P = 0.027), but no statistical difference between zone 1 and 2 (P = 1.000) and between zone 1 and 3 (P < 0.046). However, the difference in false lumen thickness between zone 1 (1.55 ± 0.51 cm) and zone 3 (1.33 ± 0.55 cm) was relatively small. Combined with the special anatomical site of zone 3, zone 1 was still preferred for catheter insertion infusion. There was no statistically significant difference between zone 2 and 3 (P = 0.080) (Table 2).

**Table 2:** True and false lumen and hematoma thickness in the three regions of the supra-aortic arch ±: Plus-minus sign indicating the tolerance or statistical margin of error of a quantity.

Variable	Brachiocephalic trunk artery area (zone 1)	Left common carotid artery area (zone 2)	Left subclavian artery area (zone 3)	F/F′ value	P-value
True lumen area (cm^2^)	6.40 ± 2.71	5.75 ± 2.13	4.85 ± 1.70	F′ = 13.298	<0.001
False lumen area (cm^2^)	7.51 ± 3.36	5.87 ± 2.45	4.37 ± 1.89	F′ = 36.152	<0.001
Total area (cm^2^)	13.91 ± 3.10	11.62 ± 2.83	9.22 ± 2.15	F′ = 80.575	<0.001
False lumen thickness (cm)	1.55 ± 0.51	1.53 ± 0.42	1.33 ± 0.55	F′ = 3.683	0.027

Preoperatively, the patient's true-false lumen type, true-false lumen area, and hematoma thickness were evaluated through imaging software to guide the position of the aortic arch catheter insertion and puncture depth during the operation. Transesophageal color Doppler ultrasound was used to determine the catheter insertion situation, which was helpful for the successful operation.

Intraoperative parameter analysis

This study aimed to evaluate the safety and efficacy of ascending aortic cannulation perfusion in 100 cases of acute DeBakey type I aortic dissection patients. Of these, 67 patients underwent the perfusion way of ascending aortic cannulation. The surgical methods used were aortic valve direct vision remodeling, ascending aortic replacement, and Sun's procedure in 35 cases; coronary artery bypass grafting, Bentall, and Sun’s procedure in 16 cases; ascending aortic replacement and Sun’s procedure in seven cases; Bentall and Sun’s procedure in three cases; aortic valve ring remodeling, ascending aortic replacement, and Sun’s procedure in two cases; tricuspid valve remodeling, aortic valve direct vision remodeling, ascending aortic replacement, and Sun's procedure in one case; coronary artery bypass grafting, aortic valve direct vision remodeling, ascending aortic replacement, and Sun's procedure in one case; aortic sinus remodeling, coronary artery bypass grafting, aortic valve direct vision remodeling, ascending aortic replacement, and Sun's procedure in one case; and aortic sinus remodeling, ascending aortic replacement, and Sun’s procedure in one case. The intraoperative extracorporeal circulation parameters were also evaluated, which included a minimum nasopharyngeal temperature of 24.95 ± 1.35°C, extracorporeal circulation time of 245.66 ± 62.72 minutes, cooling time of 89.45 ± 19.24 minutes, selective cerebral perfusion time and lower body circulatory arrest time of 32.66 ± 5.58 minutes, aortic occlusion time of 150.40 ± 39.32 minutes, rewarming time of 92.67 ± 23.96 minutes, and perfusion pressure of 175.22 ± 10.56 mmHg. No adverse events were reported during the intraoperative extracorporeal circulation, including false lumen on ascending aortic cannulation, additional peripheral vascular cannulation, intraoperative aortic arch rupture, and intraoperative death. The results indicated that the ascending aortic cannulation perfusion was a safe and effective perfusion strategy for aortic dissection surgery. Tables 3, 4 provide detailed information on the results of the study, while Figure 1 shows a graphical representation of the perfusion strategy used in the study. (Note: Sun’s procedure refers to total aortic arch replacement + in situ elephant trunk stent implantation.)

**Table 3:** Surgical methods used on 67 patients

Surgical modalities	Patients (n = 67)	%
Mitral valve plasty + open-aortic valve plasty + ascending aorta replacement + Sun's surgery	1	1.5
Coronary artery bypass + open-aortic valve plasty + ascending aortic replacement + Sun's surgery	1	1.5
Coronary artery bypass + Bentall + Sun's surgery	16	23.9
Ascending aorta replacement + Sun's surgery	7	10.4
Aortic annuloplasty + ascending aorta replacement + Sun's surgery	2	3.0
Open aortic valve plasty + ascending aorta replacement + Sun's surgery	35	52.2
Aortic sinusoplasty + coronary artery bypass + open-aortic valve plasty + ascending aorta replacement + Sun's surgery	1	1.5
Aortic sinusoplasty + ascending aorta replacement + Sun's surgery	1	1.5
Bentall + Sun's surgery	3	4.5
Total	67	100.0

**Table 4:** Intraoperative parameters ±: Plus-minus sign indicating the tolerance or statistical margin of error of a quantity.

Variable	Value
Minimum nasopharyngeal temperature (°C)	24.95 ± 1.35
Extracorporeal circulation time (min)	245.66 ± 62.72
Cooling time (min)	89.45 ± 19.24
Selective cerebral perfusion time (min)	32.66 ± 5.58
Lower body arrest (min)	32.66 ± 5.58
Aortic occlusion time (min)	150.40 ± 39.32
Rewarming time (min)	92.67 ± 23.96
Intraoperative perfusion pressure (mmHg).	175.22 ± 10.56
Insertion of a false cavity (example)	0
Additional peripheral vascular cannulation (example)	0
Intraoperative rupture of the aortic arch (example)	0
Intraoperative death (example)	0

## Discussion

The emergency surgical treatment of acute type A aortic dissection is still recommended both domestically and internationally. Cannulation strategy is an important aspect of aortic dissection surgery. Common perfusion strategies include axillary artery cannulation and infusion, femoral artery cannulation and infusion, and femoral-axillary artery combined double perfusion or other methods. However, as mentioned earlier, these perfusion strategies have many limitations, and effective intraoperative perfusion has a positive impact on the patient's surgical progress and postoperative recovery [8]. Therefore, it is essential to explore new perfusion strategies. A Japanese scholar proposed that cannulation and infusion on the ascending aorta are safe, effective, and feasible [3]. The selection of cannulation strategy is crucial in aortic dissection surgery, and aortic arch cannulation has been found to have several advantages over other perfusion strategies, including better intraoperative perfusion flow, less patient trauma, and reduced anesthesia and operation time. However, the choice of cannulation position is of utmost importance, as even a slight deviation can lead to catheter insertion into the false lumen, with potentially serious consequences. To optimize the cannulation and perfusion strategy, a comprehensive evaluation using medical imaging software was conducted, focusing on false lumen classification, zone, false lumen area, and puncture depth. The results showed that zone 1 of the aortic arch has the largest true lumen area and is therefore the best position for puncturing needle and cannulation in patients with aortic dissection undergoing aortic arch cannulation strategy. Additionally, the position of zone 1 is on the right side of the patient's front midline, making it convenient for the surgeon to puncture. This optimized strategy can lead to smoother surgery and better postoperative outcomes for patients.

To avoid complications such as aortic rupture caused by entering the false lumen during aortic arch cannulation, our center implemented several methods for judgment. These methods included puncturing under transesophageal echocardiography to ensure that the puncture needle enters the true lumen, monitoring infusion pressure during cannulation, and exploring the aortic arch after resection to confirm the location of the catheter. A total of 100 patients were enrolled in this study, of which 67 patients were subjected to aortic arch cannulation using these methods. The statistical analysis of infusion pressure during the operation showed a mean value of 175.22 ± 10.56 mmHg, indicating that the cannula was inserted into the true lumen in all 67 patients, and no extracorporeal circulation adverse events occurred during the operation [9,10].

Prior to aortic arch clamping surgery, a detailed reading of the patient's imaging data was conducted to evaluate the true and false lumen typing, zoning, true and false lumen area, and puncture depth. This information was used to guide the implementation of the aortic arch clamping surgery and aortic arch cannulation strategy. This allowed the surgeon and assistant to understand the vascular anatomy of the aorta, puncture position, cannulation angle, and depth of the patient in detail. This approach is of great clinical significance for ensuring smooth operation, patient safety during the operation, and smooth recovery after the operation.

The study showed that aortic arch cannulation for extracorporeal circulation perfusion during aortic arch clamping surgery is an effective and safe cannulation strategy. The comprehensive evaluation demonstrated that this method offers several advantages, including a perfusion flow that is more in line with human physiology, less trauma to the patient compared to axillary or femoral artery cannulation, and reduced anesthesia and operation time. The selection of the cannulation position was found to be particularly important, with zone 1 being the best position for puncturing the needle and cannulation in patients with aortic dissection who undergo an aortic arch cannulation strategy. Careful evaluation of the patient's imaging data is an important part of this approach, as it provides guidance for the implementation of aortic arch clamping surgery and aortic arch cannulation strategy.

Overall, the study highlights the importance of using aortic arch cannulation for extracorporeal circulation perfusion during aortic arch clamping surgery and the need for careful evaluation of imaging data to ensure the safety and efficacy of the procedure. The findings of this study provide valuable insights for clinicians involved in aortic arch cannulation and may contribute to the development of safer and more effective cannulation strategies.

Limitations

This study has some limitations as it is a retrospective study conducted at a single center, and the results may be influenced by the size of the study population. Therefore, a larger sample size is required for a prospective multicenter study to confirm the safety and effectiveness of the bowstringing cannulation technique.

## Conclusions

The findings of this study suggest that zone 1 in the aortic arch has the largest area and the most favorable angle for the surgeon, which aligns with the physiological characteristics and surgical practices of the surgeon. Careful analysis of CTA and accurate measurement of related parameters can provide valuable guidance for aortic arch cannulation. Overall, the study supports the use of aortic arch cannulation as a safe and effective strategy for perfusion during aortic dissection surgery.
